# Regaining enamel color quality using enamel matrix derivative

**DOI:** 10.1007/s00795-022-00346-5

**Published:** 2023-01-09

**Authors:** Hiroyuki Sugaya, Yoshihito Kurashige, Kai Suzuki, Sayaka Sakakibara, Yusuke Fujita, Syed Taufiqul Islam, Takashi Nezu, Shuichi Ito, Yoshihiro Abiko, Masato Saitoh

**Affiliations:** 1 Division of Pediatric, Dentistry School of Dentistry, Health Sciences University of Hokkaido, 1757 Kanazawa, Tobetsu, Ishikari, Hokkaido 061-0293 Japan; 2 Division of Biomaterials and Bioengineering, School of Dentistry, Health Sciences University of Hokkaido, Tobetsu, Japan; 3 Division of Dental Education Development, School of Dentistry, Health Sciences University of Hokkaido, Tobetsu, Japan; 4 Division of Oral Medicine and Pathology, Dentistry School of Dentistry, Health Sciences University of Hokkaido, Tobetsu, Japan

**Keywords:** Amelogenin, Hydroxyapatite crystals, Emdogain, Mineralization buffer, CIE L*a*b*, Hap

## Abstract

This study aimed to demonstrate and compare the accuracy of tooth shade selection due to the remineralized enamel crystal with enamel matrix derivative (EMD) in vitro. Etched enamel slices were immersed in four types of mineralization buffers for 16 h. Sodium fluoride (NaF) was added to final concentrations of 1–100 ppm with the mineralization buffer that demonstrated the highest mineralization efficiency. EMD was added to the mineralization buffer containing NaF to see if it has any remineralization capacities. The remineralized enamel crystal was analyzed by SEM and XRD. The tooth shade was evaluated by CIE L*a*b*. The results showed that, without NaF, plate-like nanocrystals were formed on the enamel surface, but with NaF, needle-like nanocrystals were formed. By adding EMD, a layer of well-compacted hydroxyapatite crystals was successfully precipitated onto the natural enamel surface. No significant differences were observed in the L* value of the mineralization surface pre-etching and after mineralization buffer containing NaF and EMD. A new method has been developed to recover the color quality of enamel, as well as to mineralize the tooth enamel by constructing hydroxyapatite crystals with mineralization buffers containing NaF and EMD on the etched tooth surface.

## Introduction

Dental enamel is the hardest mineralized tissue in the human body and is composed of more than 95% hydroxyapatite crystals by weight [1]. A sound enamel surface in the oral environment conserves the subtle balance between the demineralization and remineralization processes. However, decalcification, when dental enamel loses minerals due to various intraoral acid conditions, occurs easily. Demineralization of enamel results in visible changes such as loss of gloss and shine and an opaque and chalky-white appearance, clinically known as white spot lesions (WSLs) [2]. Mouth rinses and toothpastes with fluoride have been applied to WSLs for remineralization [3–5]. However, the fluoride applications frequently do not provide esthetic restoration of the WSLs [6].

In addition, casein phosphopeptide-amorphous calcium phosphate (CPP-ACP) paste, bioglass materials, have been attempted to promote enamel remineralization at the early stage of demineralization of the enamel surface, but regeneration of the enamel is not established [7, 8]. The remineralization outcomes and its consequences in the optical properties of enamel depend on the extension/depth of the demineralized tissue.

Regeneration of enamel provides enough hardness for the oral environment, prevention of initial caries, and improved esthetic features on the surface of the tooth. Enamel demineralization should be restored with the regenerative enamel more than the mineralization. The regenerated enamel is distinguished from the mineralization by the presence of the crystal structure of hydroxyapatite [9].

Various studies of regenerating enamel-like hydroxyapatite crystals have been performed under extreme non-physiological conditions, such as a high-temperature, high-pressure, low acidic pH, or in the presence of surfactants [10–12]. These biomimetic approaches for regenerating enamel that do not require extreme conditions would be clinically useful in pediatric, preventive, and operative dentistry. Such biomimetic methods are performed in physiological conditions using calcium phosphate buffer including amelogenin [13, 14].

Enamel formation is started by the secretion of the enamel matrix protein that promotes differentiated ameloblasts in the enamel organ of the bell stage during enamel development. Secreted amelogenin accounts for more than 90% of enamel matrix protein from ameloblasts, and enamelin or ameloblastin is present in small amounts [15, 16]. Self-assembly of amelogenin controls the oriented and elongated growth of the hydroxyapatite crystals within enamel prisms [17–19]. Use of recombinant amelogenins to form hydroxyapatite crystals has been attempted under several in vitro conditions [13, 20]. Although the recombinant amelogenins have been produced by several methods including *Escherichia coli-*based, yeast-based, and virus expression systems, no methods have been widely adopted due to uncertainty, such as involvement of its phosphorylation [21]. Moreover, the recombinant amelogenins are very expensive for clinical applications. Instead, enamel matrix derivative (EMD: Emdogain^®^, Straumann, Allschwil, Switzerland) from porcine tooth buds, consisting mainly of amelogenin fragments, has been clinically applied for periodontal regeneration. EMD could promote the remineralization of enamel in vitro [22, 23]. An electron microscopic study showed that formation of enamel crystals on the sliced enamel was induced by EMD with fluoride soaked into calcium chloride solution for 96 h [24]. This study did not examine esthetic features, which is an important finding of the regenerated enamel. The whole tooth should be used to evaluate the esthetic features of the regenerated enamel.

Therefore, we hypothesized that the hydroxyapatite crystals formed with mineralization buffer containing EMD provide esthetic remineralization. This study examined how a calcium phosphate mineralization buffer containing EMD induced mineralization on the etched enamel slices within 16 h, and how the mineralization was esthetic on the etched enamel, mimicking WSLs in the extracted whole tooth crown.

## Materials and methods

### Ethics

The study was approved by the research ethics committee of our Institution (Reference No. 2016-0010). All patients who underwent tooth extraction provided written, informed consent in accordance with the revised Declaration of Helsinki (World Medical Association Declaration of Helsinki 2008).

### Enamel slice samples

Thirty human permanent maxillary and mandibular central teeth that were extracted due to periodontal problems were chosen. The teeth were caries-free and had no coronal restoration, crack, or fractures. Extracted teeth were washed in 3% hypochlorous acid and saline solution, and 1-mm-thick slices of enamel were cut using a low-speed, precision sectioning saw (Buehler, Lake Bluff, IL, USA). The prepared enamel slices were stored in saline solution at 4 °C. The slices were surface-treated with 40% phosphoric acid (K-ETCHANT GEL, Kuraray Noritake Dental Inc., Tokyo, Japan) for 60 s and then washed in distilled water and dried, and then subsequently immersed in mineralization buffer.

### Mineralization buffers

Four mineralization buffers were prepared, solutions A, B, C, and D [25, 26].

Mineralization buffer A was prepared with 2.12 mM calcium chloride dihydrate (CaCl_2_·2H_2_O: Fujifilm Wako Pure Chemical Co., Osaka, Japan), 1.27 mM K-PO_4_, which is a mixture of potassium dihydrogenphosphate and dipotassium hydrogen phosphate (KH_2_PO_4_, K_2_HPO_4_: Kanto Chemical Co., Inc., Tokyo, Japan). Mineralization buffer B was prepared with 2.24 mM CaCl_2_·2H_2_O and 1.34 mM K-PO_4_. Mineralization buffer C was prepared with 2.35 mM CaCl_2_·2H_2_O and 1.41 mM K-PO_4_. The final pH of mineralization buffers A, B, and C was adjusted to 7.4 by 10 mM HEPES–KOH [prepared from 10 mM hydroxyethyl piperazine ethane sulfonic acid (HEPES: Nacalai Tesque, Inc., Kyoto, Japan) mixed with potassium hydroxide (KOH: Nacalai Tesque, Inc.), and 150 mM potassium chloride (KCl: Nacalai Tesque, Inc.)].

Mineralization buffer D was prepared with 2.58 mM CaCl2·2H2O, 1.55 mM KH_2_PO_4_, and 180 mM sodium chloride (NaCl); the pH was adjusted to 7.6 with 50 mM Tris–HCl.

With all mineralization buffers, pH adjustment was carried out using a pH meter (F-22: Horiba Ltd., Kyoto, Japan). All of the mineralization buffers used in the present study had a Ca/P molar ratio of 1.67; the degrees of solution saturations with respect to HAp were (A) 2.58 × 10^7^, (B) 3.85 × 10^7^, (C) 5.50 × 10^7^, and (D) 8.32 × 10^7^ [25].

### Remineralization of enamel slices

Enamel slices were immersed for 16 h in mineralization buffer A, B, C, or D (*n* = each 10 slices). After immersion, the slices were held on copper plates (*ϕ*13.4 mm) covered with electrically conductive double-sided tape for carbon deposition. Following this, the surface of the enamel slices was observed using a field emission scanning electron microscope (SEM: JSM-7800F, JEOL Ltd., Tokyo, Japan) to determine which mineralization buffer showed the best mineralization efficiency.

### Determination of the amount of fluoride

Sodium fluoride (NaF: Kanto Chemical Co., Inc.) was added to final concentrations of 1, 10, and 100 ppmF^−^ with the mineralization buffer that demonstrated the highest mineralization efficiency. Enamel slices were then immersed for 16 h in the mineralization buffer containing NaF at each of these concentrations, and assessed under the SEM.

### Determination of the amount of EMD

Emdogain^®^ (EMD: Straumann, Allschwil, Switzerland) was added at concentrations of 0.05%, 0.1%, 0.5%, and 1% to the mineralization buffer containing NaF, and the enamel slices were immersed and assessed under the SEM. Since EMD contains 6% propylene glycol alginate (PGA), 6% PGA (Wako Pure Chemical Co., Tokyo, Japan) was added to the mineralization buffer containing NaF as the control.

### X-ray diffraction analysis of the remineralized enamel surface

Enamel slices were prepared for analysis using an X-ray diffraction (XRD) device (RINT200: Rigaku, Tokyo, Japan; Cu X-ray source, output: 40 kV at 200 mA) after being immersed in mineralization buffer alone, mineralization buffer containing NaF, and mineralization buffer containing NaF and EMD. The two-dimensional X-ray diffraction patterns were used to identify the deposited crystals and to study their crystallinity. Enamel slices without etching and enamel slices after surface treatment with 40% phosphoric acid for 60 s were used as controls. The hydroxyapatite (HAp) patterns were examined to identify any differences between the groups. For the intensity ratio of the samples, the ICDD PDF values were used (Ca_5_(PO_4_)_3_OH: 00-034-0010: Quality S, Ca_10_(PO_4_)_6_OH_2_: 01-072-1243: Quality I).

### Color tone changes in the tooth crown

Extracted teeth were first rinsed with 3% hypochlorous acid and saline solution, the entire enamel surface-treated with 40% phosphoric acid for 60 s, and then immersed for 16 h in mineralization buffer alone, mineralization buffer containing NaF, and mineralization buffer containing NaF and EMD. All teeth were photographed using a digital camera (D5200, Nikon, Tokyo, Japan), 100-mm macro lens and speed light ring flash in a windowless room under standardized setting [exposure, 1/200 s; aper-ture, f/11; white balance flash at a fixed object-lens distance (22 cm)] and recorded in TIFF file format. To standardize color and the exact location each time a photograph was taken with a Casmatch (Bear Medic Corporation, Tokyo, Japan) placed near the tooth examined in Fig. 7b. The captured images were recorded as TIFF data, and colorimetry was performed in Photoshop CC (Adobe Inc., San Jose, CA, USA). Color correction was carried out using black, gray, and white, and then levels were corrected in Photoshop [27, 28]. The values were determined from 10 randomly selected sites on the enamel surface. Colors were classified using the L*a*b* color system [Commission Internationale de l'Eclairage (CIE) 1976], where L* is the brightness index and a* and b* are indices of perceived chromaticity. The mean values of L*, a*, and b* were measured. For color tone correction separately from the Casmatch, each tooth was partially filled with A2 composite resin (Kuraray Noritake Dental Inc., Tokyo, Japan) as a control [29].

The CIELAB system systematically arranges colors in a way that correlates with visual perception, allowing them to be shown as a specific color. Higher values of L* indicate greater brightness and whiter colors, higher values of a* indicate more red in the positive (+) direction and more green in the negative (−) direction, and higher values of b* indicate more yellow in the positive direction and more blue in the negative direction. Changes in color over time were determined by measuring the values pre-etching (*l*_p_*, *a*_p_*, *b*_p_*), after etching (*l*_b_*, *a*_b_*, *b*_b_*), and after immersion in mineralization buffer (*l*_q_*, *a*_q_*, *b*_q_*). Color differences (Δ*Eab*) pre-etching and after immersion in mineralization buffer were compared by calculating the values of Δ*L*, Δ*a*, and Δ*b* at each of these two times. 10 sites were randomly selected in each tooth for the calculation.

Difference in color was calculated by the following formula:

$$\Delta E = \left\{ {\left( {l_p * - l_q *} \right)^2 + \, \left( {a_p * - a_q *} \right)^2 + \, \left( {b_p * - b_q *} \right)^2 } \right\}^{1/2} .$$

### Statistical analysis

From the colorimetric results, the values of L*, a*, and b* pre-etching, after etching and after immersion in mineralization buffer were analyzed using IBM SPSS Statistics version 21 (IBM, Armonk, NY, USA). Differences in the values were tested using one-way analysis of variance (ANOVA) followed by Scheffe’s test, with *p* < 0.05 taken to be significant.

## Results

### Mineralization buffer

**Fig. 1:**
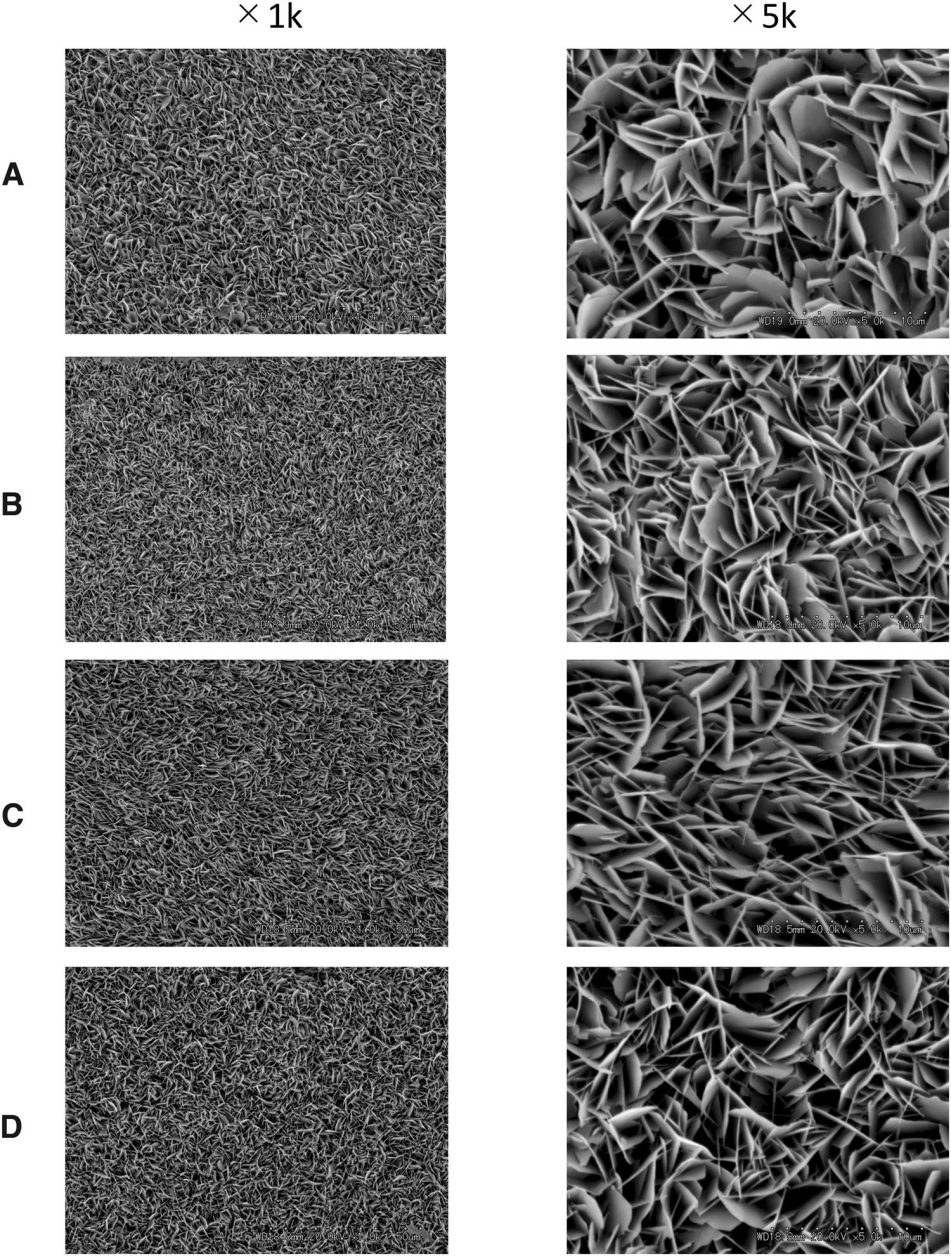
SEM images of the demineralized enamel surface after immersion in mineralization buffer. **A** Mineralization buffer A, **B** mineralization buffer B, **C** mineralization buffer C, **D** mineralization buffer D. Mineralization buffers A, B, and C formed denser plate-like structures with higher Ca/P ratios than mineralization buffer D. Images were taken at 1 k and 5 k magnifications

SEM was used to assess remineralization after enamel slices were immersed in mineralization buffers A, B, C, and D for 16 h. Figure 1 shows the structures deposited on the surface of the enamel slices by different mineralization buffers. Plate-like structures were found in all samples with mineralization buffers A, B, C, and D. With mineralization buffers A, B, and C, the plate-like structures were denser with higher HAp ratios. Compared to A, B, and C, the plate-like structures were sparse with mineralization buffer D. The calcified precipitates appeared in all mineralization buffer at 16 h, and amount of the precipitation in C was lowest among the mineralization buffers. Therefore, mineralization buffer C was selected for use in the subsequent experiments.

### Determination of the amount of fluoride

**Fig. 2:**
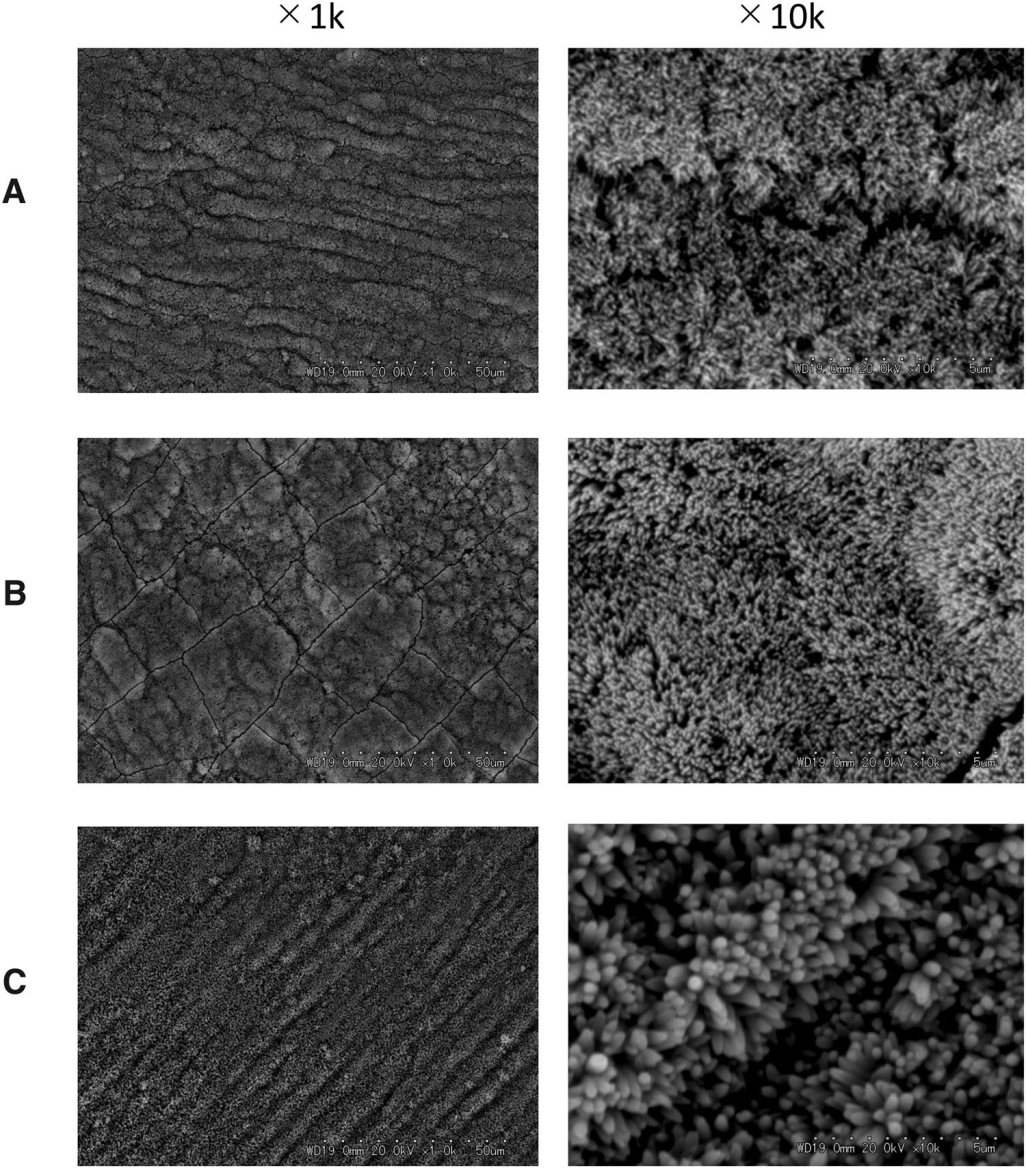
SEM images of the demineralized enamel surface after immersion in mineralization buffer C. **A** Mineralization buffer C + 1 ppm NaF, **B** mineralization buffer C + 10 ppm NaF, **C** mineralization buffer C + 100 ppm NaF. The addition of NaF transformed the plate-like remineralization structures into needle-like structures. At higher fluoride concentrations, the needle-like depositions were thicker. Images were taken at 1 k and 10 k magnifications

Sodium fluoride was added to mineralization buffer C to final concentrations of 1, 10, and 100 ppm, and remineralization procedures were carried out. The plate-like remineralized structures were transformed into needle-like structures as a result of the addition of NaF. The needle-like depositions were thicker at higher fluoride concentrations (Fig. 2). On the basis of these results, a fluoride concentration of 100 ppm was selected for use in the subsequent experiments.

### Determination of the amount of EMD

**Fig. 3:**
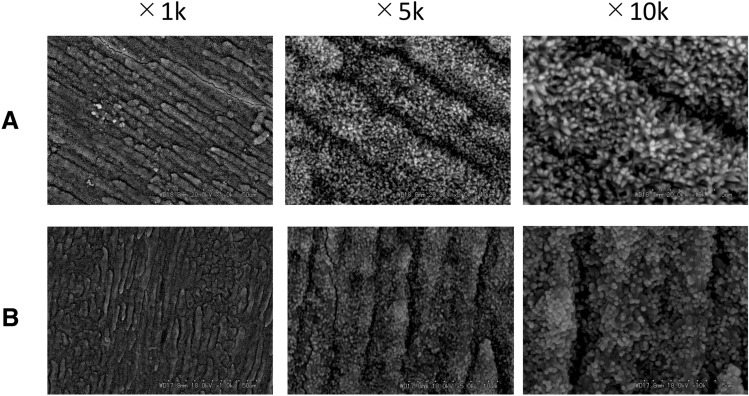
SEM images of the demineralized enamel surface after immersion in mineralization buffer C. **A** Mineralization buffer C + 100 ppm NaF, **B** mineralization buffer C + 100 ppm NaF + 6% PGA. There are no differences between A and B in the deposited crystals. Images were taken at 1 k, 5 k and 10 k magnifications

EMD (0.05%, 0.1%, 0.5%, and 1%) or 6% PGA (control) was added to the NaF-containing mineralization buffer, and mineralization was evaluated by SEM. No differences in the deposited crystals were observed between the mineralization buffer containing-NaF and the mineralization buffer containing-NaF with 6% PGA (Fig. 3).

**Fig. 4:**
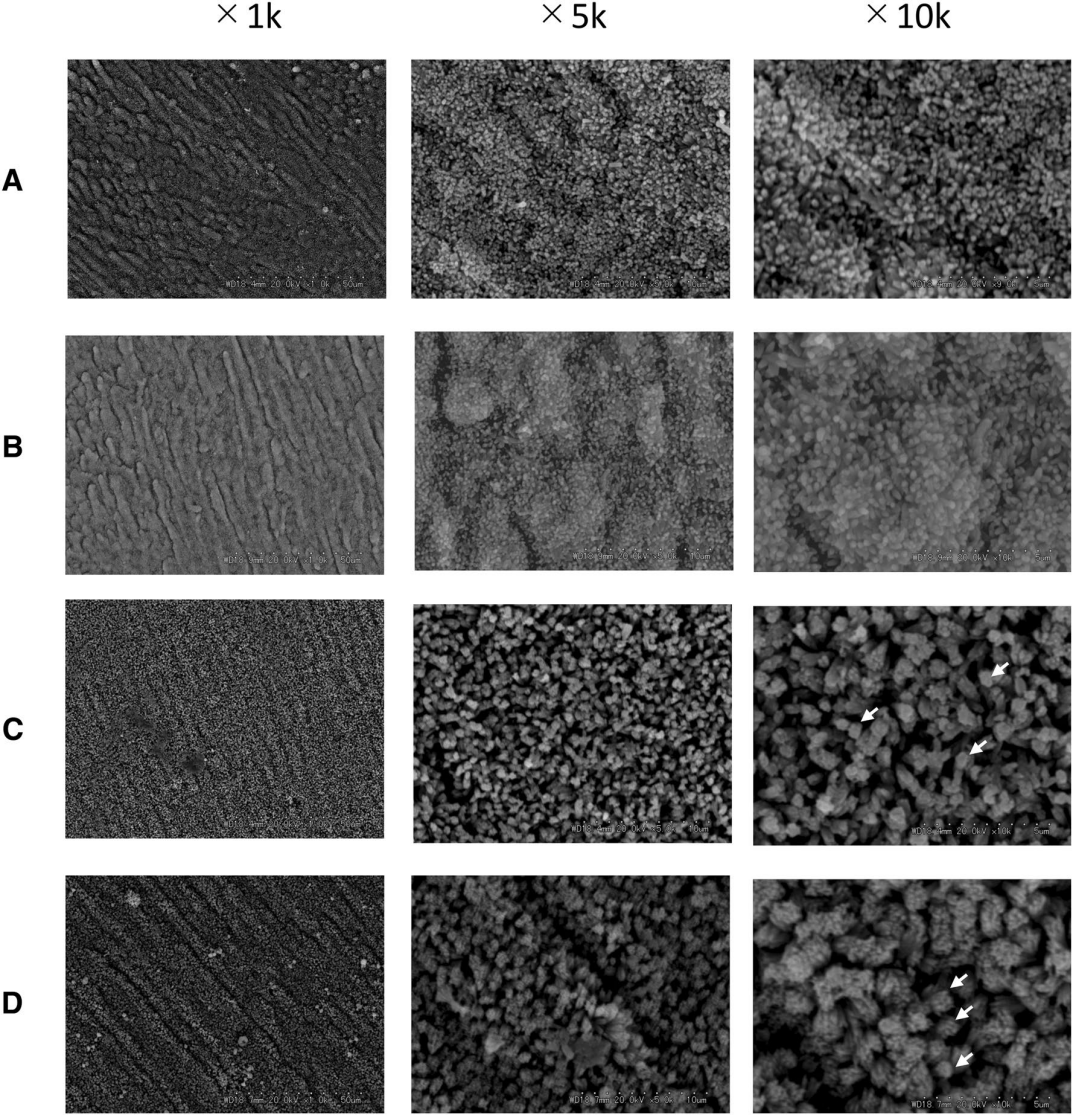
SEM images of the crystals formed on demineralized enamel surface after remineralization by mineralization buffer C with NaF and EMD added. **A** Mineralization buffer C + 100 ppm NaF + 0.05% EMD, **B** mineralization buffer C + 100 ppm NaF + 0.1% EMD, **C** mineralization buffer C + 100 ppm NaF + 0.5% EMD, **D** mineralization buffer C + 100 ppm NaF + 1% EMD. In A and B, needle-like deposits formed, and in C and D, needle-like deposits aggregated to form columnar shapes. These aggregations are much larger at higher EMD concentrations. Images were taken at 1 k, 5 k, and 10 k magnifications. The arrows indicate columnar structures

Needle-like deposits were formed with the mineralization buffer containing-NaF with 0.05% and 0.1% EMD. In contrast, the needle-like deposits aggregated to form columnar shapes with the mineralization buffer containing-NaF with 0.5% and 1% EMD. At higher EMD concentrations, these aggregated columnar structures were larger (Fig. 4).

### XRD analysis of the remineralized enamel surface

**Fig. 5:**
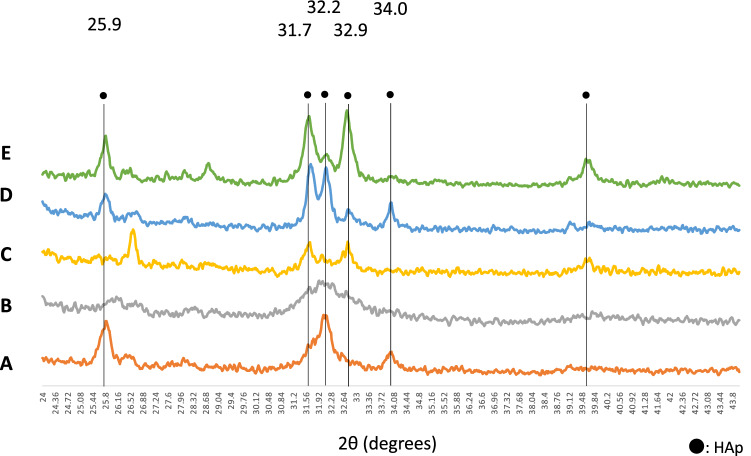
Two-dimensional X-ray diffraction patterns of enamels immersed in various mineralization solutions. **A** Normal tooth (pre-etching), **B** after etching, **C** after mineralization buffer C alone, **D** after mineralization buffer C + 100 ppm NaF, **E** after mineralization buffer C + 100 ppm NaF + 1% EMD. HAp diffraction peaks can be seen in A at 2*θ* = 25.9°, 32.2°, and 34.0°. In B, the intensity of the peaks is less, and the width of the peaks is wider. HAp peaks are found at 2*θ* = 25.9°, 31.7°, 32.2°, 32.9°, and 34.0° in mineralization buffer C alone (**C**), mineralization buffer containing NaF (**D**), and mineralization buffer containing NaF and EMD (**E**). In addition to those pre-etching of enamel, several additional high-intensity peaks are visible here

Figure 5 shows the two-dimensional X-ray diffraction XRD patterns that were obtained. In comparison to the enamel slices before and after etching, different peaks were found on the enamel surface depending on the immersion conditions.

While clear HAp diffraction peaks were visible at 2*θ* = 25.9°, 32.2°, and 34.0° in the enamel slices pre-etching, the intensity of the peaks was less, and the width of the peaks was greater in enamel slices after etching. With the enamel slices after immersion in mineralization buffer alone, mineralization buffer containing-NaF, and mineralization buffer containing-NaF and EMD,: HAp peaks were found at 2*θ* = 25.9°, 31.7°, 32.2°, 32.9°, and 34.0°. With the mineralization buffer, high-intensity HAp peaks not present in enamel after etching were found at 2*θ* = 31.7° and 32.9°. With mineralization buffer containing-NaF, high-intensity HAp peaks were found at 2*θ* = 31.7°, 32.2°, and 34.0°. With mineralization buffer containing-NaF and EMD, high-intensity HAp peaks were found at 2*θ* = 31.7°, 32.2°, and 32.9°.

### Color tone changes in the tooth crown

**Fig. 6:**
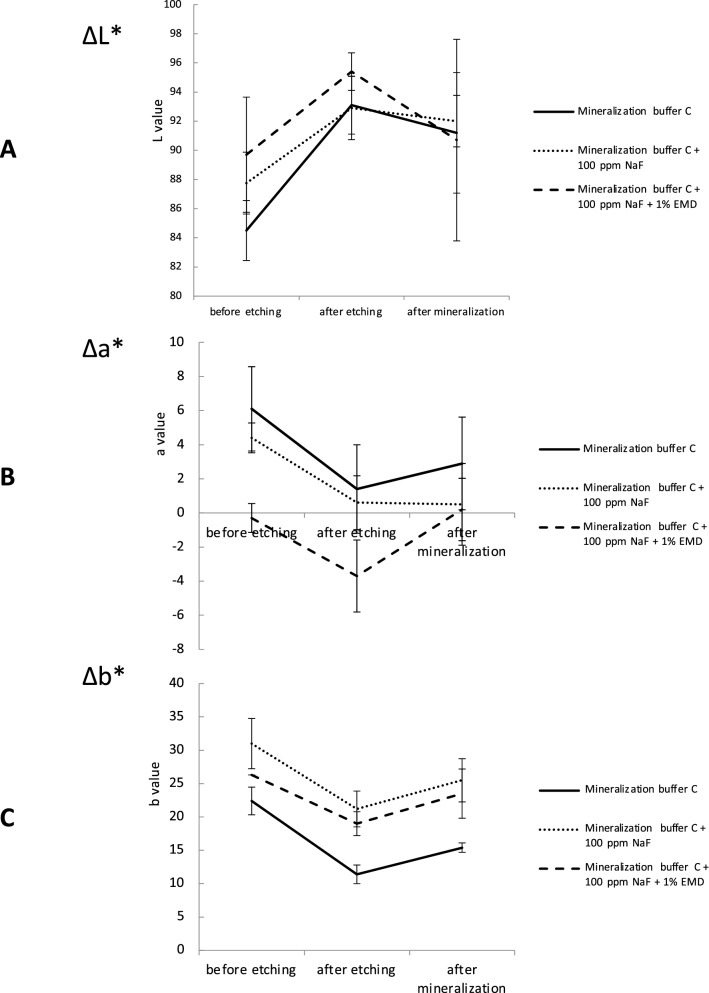
Mean tooth surface ΔL*, Δa*, and Δb* values pre-etching, after etching, and after remineralization with mineralization buffer C. **A** ΔL* values are higher in mineralization buffer C added with 100 ppm NaF and 1% EMD. **B** Δa* is shown to be the least in mineralization buffer C added with 100 ppm NaF and 1% EMD. **C** Δb* is lowest in mineralization buffer C added with 100 ppm NaF and 1% EMD

**Fig. 7:**
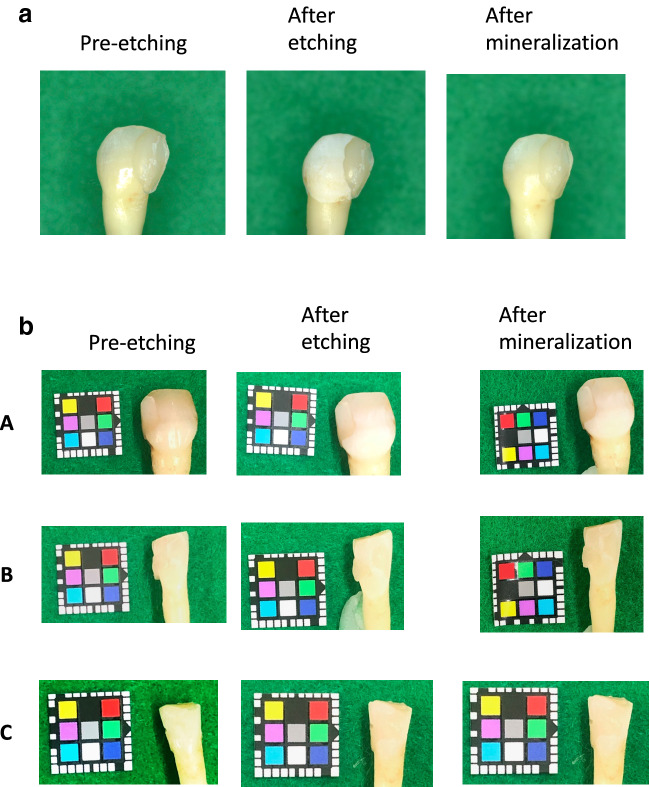
**a** The visual differences of immersion in mineralization buffer C + 100 ppm NaF + 1% EMD. **b** The visual differences in tooth enamel were evaluated using the CIE L*a*b* system as follows: **A** immersion in mineralization buffer C, **B** immersion in mineralization buffer C + 100 ppm NaF, **C** immersion in mineralization buffer C + 100 ppm NaF + 1% EMD

Photographs of immersed teeth and changes in the L*a*b* values are shown in Figs. 6 and 7. With the mineralization buffer alone, the mean L* value pre-etching was 84.5, after etching was 93.1, and after immersion was 91.2; the difference with respect to the value pre-etching was significant after etching (*p* = 0.001) and after immersion (*p* = 0.04). The mean a* value pre-etching was 6.1, after etching was 1.4, and after immersion was 2.9, with no significant difference after etching (*p* = 0.4) or after immersion (*p* = 0.9). The mean b* value pre-etching was 22.4, after etching was 11.4, and after immersion was 15.4; the pre-etching value was significant after etching (*p* = 0.00) and after immersion (*p* = 0.025).

With the mineralization buffer containing-NaF, the mean L* value pre-etching was 87.7, after etching was 92.9, and after immersion was 92.0, the difference was significant after etching (*p* = 0.002) and after immersion (*p* = 0.048). The mean a* value pre-etching was 4.4, after etching was 0.6, and after immersion was 0.5; the difference was significant after etching (*p* = 0.048) and after immersion (*p* = 0.037). The mean b* value pre-etching was 31.0, after etching was 21.2, and after immersion was 25.5; the pre-etching value was significant after etching (*p* = 0.000) and after immersion (*p* = 0.000).

With the mineralization buffer containing-NaF and EMD, the mean L* value pre-etching was 89.7, after etching was 95.4, and after immersion was 89.9; the difference was significant after etching (*p* = 0.000), but not significant after immersion (*p* = 1). The mean a* value pre-etching was − 0.8, after etching was − 3.7, and after immersion was 0.2; the difference was significant after etching (*p* = 0.000) and after immersion (*p* = 0.000). The mean b* value pre-etching was 25.7, after etching was 22.5, and after immersion was 23.7; the pre-etching value was significant after etching (*p* = 0.000), but not significant after immersion (*p* = 0.716).

The visual differences in tooth enamel were evaluated using the CIE L*a*b* system after being immersed in mineralization buffer C, mineralization buffer C + 100 ppm NaF, and mineralization buffer C + 100 ppm NaF + 1% EMD (Fig. 7). The value of Δ*E* from pre-etching to after immersion was 52.06 with mineralization buffer alone, 31.8 with mineralization buffer containing NaF, and 3.525 with mineralization buffer containing NaF and EMD.

## Discussion

The present study demonstrated that calcium phosphate containing NaF and EMD successfully precipitated hydroxyapatite crystals to cause the whole surface of natural enamel to be covered with the crystals for 16 h. No significant differences in the mineralization surface’s L* were observed pre-and after etching. The present method was developed to recover the shiny appearance of enamel, as well as to mineralize the tooth enamel.

Enamel slices were immersed in mineralization buffers A, B, C, and D for 16 h, and deposition of plate-like structures was observed in all samples. These deposits have been identified as octacalcium phosphate (OCP), and they may be a precursor of hydroxyapatite (HAp) [30–33]. In addition, dense deposits were found with all of the mineralization buffers, suggesting that OCP deposition is affected by the degree of HAp saturation.

Addition of NaF to the mineralization buffer caused the remineralization to take on a needle-like structure. The needle-like structures became thicker at higher fluoride concentrations. Enamel HAp exhibits a needle-like structure, and the similar structure in the enamel probably possess similar mechanical properties [34, 35]. At 100 ppm NaF, the needle-like structures had a thick structure, and some hexagonal columnar structures were observed, whereas at 1 ppm and 10 ppm, the needle-like structures were thin, and no clear hexagonal columnar structures were observed. Fluoride had an epitaxial action to suppress the plate-like growth of OCP and promote needle-like growth, and *F*-might acts on the surface of the plates that make up the plate-like structure, resulting in loss of the surface and the formation of needle-like structures [36]. This suggestion may be exemplified by the present data that showed that higher fluoride concentrations induced thicker needle-like structures.

EMD consists of proteins extracted from 6-month-old porcine molar and premolar germs, containing more than 95 wt% amelogenin fragment, and it is used clinically for induction and regeneration of alveolar bone [37]. The EMD always contains 6% PGA as the solvent. In the present results, no change in remineralization between with or without PGA in remineralization buffer was observed, suggesting that PGA itself has no action with respect to the induction or the shape of the mineral deposits. Formation of enamel prism-like crystals on the sliced enamel was induced by EMD with calcium chloride agarose hydrogel containing 500 ppm fluoride for 96 h [24]. Since the depositions of HAp precursor-like structures were observed on the enamel slices with in the mineralization for 16 h, it was we observed how addition of EMD to NaF induced affected mineralization and their structures on the enamel slice soaked in the buffer for 16 h. The mineralization experiments were performed with the addition of 0.05%, 0.1%, 0.5%, and 1% EMD for 16 h. With 0.05% and 0.1% EMD added to mineralization buffer containing NaF, needle-like deposits formed in the same way as with mineralization buffer containing NaF. With 0.5% and 1.0% EMD added to mineralization buffer containing NaF, the needle-like structures were observed to aggregate into columnar deposits, and the columnar structures were thicker at higher EMD concentrations. As stated above, fluoride could promote to form the needle-like structures. The EMD might provide columnar structures by aggregation of the needle-like structures. Amelogenin preferentially absorbed on the face of hexagonal apatite crystals to induce the columnar structure [38, 39]. Amelogenin, a main component in the EMD, may contribute formation of the columnar structures. Natural enamel consists of tight assemble of the columnar structures, whereas the columnal structures were loosely packed in the present experiments. The higher concentrations of EMD provided thinner columnal structures in this study. Recombinant amelogenin induced tightly packed columnal structures in vitro [40, 41]. Therefore, purified amalogenin may provide enamel prism-like structure better than EMD.

XRD intensity is considered a quantitative estimation and of the site occupancy of the crystal layer [42–44]. In the present results, immersion in mineralization buffer gave clear peaks at 2*θ* = 31.7° and 32.9°, indicating that there was formation of deposits, but from the lack of change in the intensity, it appears that the site occupancy of the deposits was low. With mineralization buffer containing NaF, clear peaks were found at 2*θ* = 31.7°, 32.2°, and 34.0°, with an increase in intensity and a decrease in peak width, suggesting a high site occupancy in the deposit and increased crystallinity. With mineralization buffer containing NaF and EMD, clear peaks were found at 2*θ* = 31.7°, 32.2°, and 32.9°, with an increase in intensity and a decrease in peak width, thus also suggesting a high site occupancy in the deposit and increased crystallinity. With the enamel slices pre-etching, an HAp peak was observed at 2*θ* = 25.9°. Peak width is used to evaluate crystallinity, and the decreased peak width of the immersed enamel slices in the present results therefore suggests high crystallinity of the deposits [45]. Amelogenin is believed to control the elongation, morphology [46, 47], and regular arrangement of crystal structures in tooth mineralization [48], and therefore the present results indicate that EMD assisted in crystal construction and improved the crystallinity.

The mean L* value increased significantly after etching. A higher value of L* indicates a brighter, whiter color, and the increase in L* was therefore the result of demineralization of the surface layer of the enamel by 40% phosphoric acid. After immersion in mineralization buffer and in mineralization buffer containing NaF, the value of L* increased significantly with respect to the value pre-etching, but there was no significant difference in L* after immersion in mineralization buffer containing NaF and EMD with respect to the value pre-etching. SEM observation showed that the deposit formed a columnar structure with mineralization buffer containing NaF and EMD, suggesting that this columnar structure affects the color tone.

The value of Δ*E* from pre-etching to after immersion was 52.06 with mineralization buffer, 32.9 with mineralization buffer containing NaF, and 3.525 with mineralization buffer containing NaF and EMD. Most studies set the proposed acceptance limit for color matching to 3.7, beyond which the differences are clinically visible [49–51]. In the present study, Δ*E* was 3.525 with mineralization buffer containing NaF and EMD, suggesting that the appearance of the enamel was restored to the acceptable levels as pre-etching. It is, however, not known that this method is the best for the color tone among the methods previously reported. Further investigations are needed to clarify about it.

In conclusion, the present study demonstrated that calcium phosphate containing NaF and EMD successfully precipitated hydroxyapatite crystals onto the natural enamel surface in 16 h. These mineralization structures that exhibited HAp structures at 2*θ* = 25.9° might restore the color tone of the enamel after demineralization. The present method may provide a new method to regain shiny enamel after initial demineralization of enamel.

## Data Availability

The data that support the findings of this study are available from the corresponding author, M Saitoh, upon reasonable request.
