# Correction: Regaining enamel color quality using enamel matrix derivative

**DOI:** 10.1007/s00795-023-00369-6

**Published:** 2023-08-07

**Authors:** Hiroyuki Sugaya, Yoshihito Kurashige, Kai Suzuki, Sayaka Sakakibara, Yusuke Fujita, Syed Taufiqul Islam, Takashi Nezu, Shuichi Ito, Yoshihiro Abiko, Masato Saitoh

**Affiliations:** 1 Division of Pediatric, Dentistry School of Dentistry, Health Sciences University of Hokkaido, 1757 Kanazawa, Tobetsu, Ishikari, Hokkaido 061-0293 Japan; 2 Division of Biomaterials and Bioengineering, School of Dentistry, Health Sciences University of Hokkaido, Tobetsu, Japan; 3 Division of Dental Education Development, School of Dentistry, Health Sciences University of Hokkaido, Tobetsu, Japan; 4 Division of Oral Medicine and Pathology, Dentistry School of Dentistry, Health Sciences University of Hokkaido, Tobetsu, Japan

**Correction: Medical Molecular Morphology (2023) 56:116–127** 10.1007/s00795-022-00346-5

In the original publication of the article, the last sentence “All of the mineralization buffers used in the present study had a Ca/P molar ratio of 1.67; the degrees of solution saturations with respect to HAp were (A) 2.58 × 107, (B) 3.85 × 107, (C) 5.50 × 107, and (D) 8.32 × 107 [25].” should be deleted in “Materials and methods”, under the subheading “Mineralization buffers” in the last paragraph in page 117.

